# Profile and professional expectations of medical students from 11 Latin American countries: the Red-LIRHUS project

**DOI:** 10.1186/s13104-017-2479-y

**Published:** 2017-04-20

**Authors:** Percy Mayta-Tristán, Reneé Pereyra-Elías, Juan José Montenegro-Idrogo, Christian R. Mejia, Fiorella Inga-Berrospi, Edward Mezones-Holguín, Red-LIRHUS (Grupo Colaborativo Latinoamericano para la Investigación en Recursos Humanos en Salud), Adriana Sanchez-Pozo, Juan Pablo Cardozo-López, Silvia Luizaga-Panozo, Rhanniel Theodorus-Villar, Milisen Vidal, Roxana Sepúlveda-Morales, Gabriel Abudinén A, Patricio Alfaro-Toloza, Romina Olmos-de-Aguilera, Juan Pablo Sánchez-Gonzáles, Ignacio Navarro-Brito, Jairo A. Sierra-Avendaño, Fabián Carreño, Jennifer Gomez-Alhach, Francisco Bonilla-Escobar, Omar-Javier Calixto, Álvaro Mondragón-Cardona, Jorge Ortega-Arias, Laura Agudelo-Cifuentes, Kevin Acosta, Martha Ospina, Germán D. Londoño Ruíz, Andrés Felipe Quimbayo-Cifuentes, Ingrid Benítez-Ortega, Christian D. Valverde-Lozano, Jorge Barrezueta-Fernández, Luis Cerna-Urrutia, Geovanna Moya, Gilberto Yescas, Maribel Vizárraga-León, Erick Gutierrez-Quezada, Rita Azucas-Peralta, Roy R. Vasquez-Sullca, José Antonio Grandez-Urbina, Franco León-Jiménez, Cristian Diaz-Velez, John Cabrera-Enriquez, Fiorella Inga-Berrospi, Katia Montalván-Muñoz, Oscar Moreno-Loaiza, María Molina-Torres, Johana Ávila-Figueroa, Martha Torres-Dextre, Nelson Purizaca-Rosillo, Omar Raraz-Vidal, Diego Ernesto Valencia-Chambi, Mónica Alfonso, Diego Lizarzaburu-Castagnino, Cesar Mogollón, Julio Maquera-Afaray, Mario Johnson-Franco, Gerardo Florián-Gómez, Jimmy Jeison Castro, Erik J. Jhonston, Miguel Odar-Sampé, Gelsing Richard Vásquez-García, Kelly Herencia-Anaya, Felix Ancalli-Calizaya, Lizeth Guzmán, Carlos E. Muñoz-Medina, Manuel A. Rodríguez, Adrián DaSilva-DeAbreu

**Affiliations:** 1 Escuela de Medicina, Universidad Peruana de Ciencias Aplicadas, Lima, Peru; 2 Escuela de Postgrado, Universidad Privada Antenor Orrego, Trujillo, Peru; 3 Dirección de Investigación y Desarrollo, Universidad Científica del Sur, Av. Brasil 2169 Dpto 802, Jesús María, Lima, Peru; 4 Facultad de Medicina, Universidad Nacional Mayor de San Marcos, Lima, Peru; 5 Escuela de Medicina Humana, Universidad Continental, Huancayo, Peru

**Keywords:** Human resources, Motivation, Primary Health Care, Medical education, Latin America

## Abstract

**Background:**

Latin America is undergoing a human resource crisis in health care in terms of labor shortage, misdistribution and poor orientation to primary care. Workforce data are needed to inform the planning of long-term strategies to address this problem. This study aimed to evaluate the academic and motivational profile, as well as the professional expectations, of Latin American medical students.

**Results:**

We conducted an observational, cross-sectional, multi-country study evaluating medical students from 11 Spanish-speaking countries in 2011–2012. Motivations to study medicine, migration intentions, intent to enter postgraduate programs, and perceptions regarding primary care were evaluated via a self-administered questionnaire. Outcomes were measured with pilot-tested questions and previously validated scales. A total of 11,072 valid surveys from 63 medical schools were gathered and analyzed.

**Conclusions:**

This study describes the profile and expectations of the future workforce being trained in Latin America. The obtained information will be useful for governments and universities in planning strategies to improve their current state of affairs regarding human resources for health care professions.

**Electronic supplementary material:**

The online version of this article (doi:10.1186/s13104-017-2479-y) contains supplementary material, which is available to authorized users.

## Background

Adequately trained and readily available human resources are needed for a broad range of applications to improve public health [1]. Over the years, evidence has revealed how health career-related migration to richer countries has hollowed out the human resources of developing countries [1–4]. Imbalances have also been found in workforce density within nations, favoring urban over rural areas [1, 3]. Furthermore, few professionals are found working in primary care settings in the less-advantaged areas [5, 6].

A great majority of the world still faces this crisis, with varying local circumstances and magnitudes [5]; and Latin America is no exception [7].

A number of factors have been shown to influence professional decisions of health personnel. Personal characteristics [8, 9], academic and motivational profile [10], future expectations [11, 12] and contextual working and living conditions [13–16] steer the workforce after training to their future work destinations.

Diversity regarding academic backgrounds, age at university admission, curriculums and local health systems make Latin American students a population of special interest. Additionally, there is a paucity of workforce data on the region [17, 18], even though such data are vital for planning long-term strategies regarding universal health care for the population [19, 20]. Efforts must therefore be focused on generating evidence to improve these circumstances.

## Methods

### Aims

The present study aimed to evaluate the academic and motivational profile, as well as the professional expectations, of Latin American medical students. The specific objectives were

1. Describe medical students’ motivations for choosing a career in medicine.
2. Describe the academic profile of medical students.
3. Estimate the frequencies of internal and external migration intentions of medical students, and the associated factors.
4. Evaluate medical students’ perceptions on working in a primary care setting.
5. Evaluate medical students’ personal, financial and professional expectations.

### Design

Our Collaborative Working Group for the Research of Human Resources for Health, Red-LIRHUS (Grupo Colaborativo Latinoamericano para la Investigación en Recursos Humanos en Salud) performed a cross-sectional, multi-country study in Latin America.

### Settings and participants

The study subjects were medical students from 11 Spanish-speaking Latin American countries. Our group included 63 medical schools from Bolivia, Chile, Colombia, Costa Rica, Ecuador, El Salvador, Honduras, Mexico, Paraguay, Peru and Venezuela. Medical schools from Argentina, Cuba, Guatemala, Nicaragua, Panama and Uruguay also initially joined the project but eventually departed; they were not included in the total data counts.

We aimed to make the assessment as varied as possible regarding subjects’ inclusion, targeting at least one university from each country’s capital city and one from outside it, as well as one university with public and one with private funding (Table 1). We have provided an estimated number of the existing medical schools by country, according to the World Directory of Medical Schools [21]. While this information may not be completely up-to-date, it provides a fair approximation of the number of schools.

**Table 1:** Universities participating in the study: profile and professional expectations of medical students from 11 Latin American countries

Country	Medical schools							
	Total no.^a^	No. included^b^	Funding		Location		Medical students	
			Private	Public	Capital city	Provinces	Surveys gathered	Surveys valid
Bolivia	19	4	2	2	1	3	1749	1618
Chile	19	6	3	3	0	6	611	606
Colombia	50	11	6	5	1	10	1482	1423
Costa Rica	8	1	1	0	1	0	149	148
Ecuador	23	1	0	1	0	1	1270	1174
El Salvador	6	1	1	0	1	0	94	94
Honduras	1	1	0	1	1	0	1011	990
Mexico	83	2	0	2	0	2	237	201
Paraguay	8	1	0	1	1	0	164	156
Peru	31	31	15	16	7	24	3940	3768
Venezuela	11	4	0	4	2	2	933	894
Total	259	63	28	35	15	48	11,640	11,072

^a^ Total number of medical schools by country according to the World Directory of Medical Schools [21]. This list might not be up-to-date

^b^ Number of medical schools included in the study

**Fig. 1:**
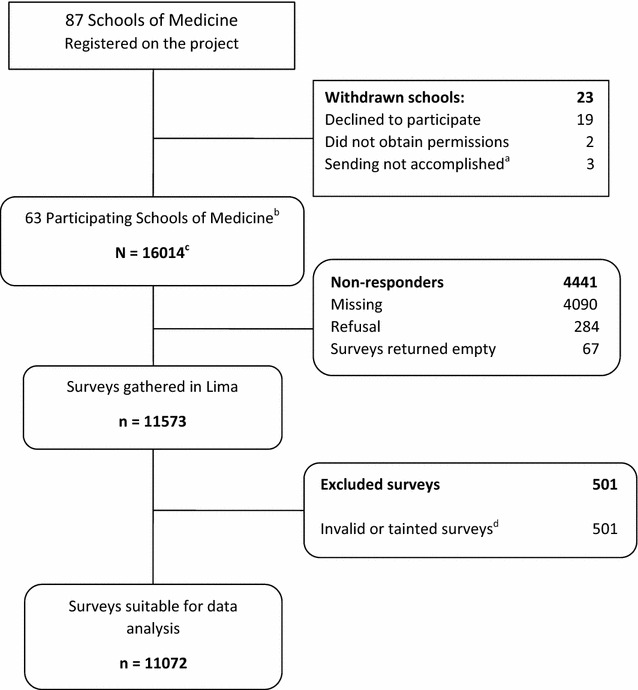
Latin American multicountry study evaluating the profile and professional expectations of physicians in training.^a^ Data collection was carried out in three medical schools, however, the shipping to Lima was not successful.^b^ Sixty three Schools of Medicine from 61 Universities given that the Universidad Central de Venezuela (Venezuela) and the Universidad de San Martín de Porres (Peru) have a subsidiary School besides the main.^c^ Total estimate of Medical Students from first and fifth year in participant schools.^d^ Surveys declared as invalidly or inappropriately fulfilled after revision

Medical school duration ranges 5–8 years. We evaluated students in their first or fifth year to compare the characteristics of students at the beginning and near the end of their course. We excluded students who refused to take part in the survey and those who completed it inappropriately or incongruously (Fig. 1). Response rates varied widely, from 59.6 to 100%, mainly due to missing study subjects. The proportion of non-responders was similar between public and private universities, but slightly higher in fifth-year students. Unfortunately, we lack accurate information about the characteristics of non-responding participants.

## Recruitment of researchers and study subjects

### Researchers

As our study subjects were medical students, we decided to involve them as the primary local-level researchers. We recruited them in two different ways. First, we made a public announcement at the Annual Medical Students’ Meeting of the Latin American Federation of Medical Students’ Scientific Societies (Federación Latinoamericana de Sociedades Científicas de Estudiantes de Medicina—FELSOCEM) in Asunción, Paraguay [22, 23].

From that, we enrolled around 30 investigators. We also recruited through Facebook by posting on the university pages of Medical Students’ Scientific Societies and the International Federation of Medical Students’ Association [24]. This strategy led to a total of 90 researchers enrolled, including medical students and physicians (who were also involved in the societies while in training), who officially became part of the project. They represented 87 schools of medicine from 17 Latin American countries (one participant for each institution, and two in exceptional cases). Ultimately, 63 researchers from 63 medical schools completed the study. Fifty-five were physicians in training, while only eight were graduated physicians. Communication and coordination during the entire research process was primarily performed via Facebook. A “Closed group” was created that added the local researchers as members. The official documents (study protocol, survey, specific procedures guidelines for each stage of the project, ethical approvals and letters of endorsement for researchers to present the study to local authorities) were uploaded in this space. All discussions and uncertainties were solved through member interaction to ensure a standardized data collection process [24]. Authorship credits were proposed at the beginning of the study.

### Data collection

**Fig. 2:**
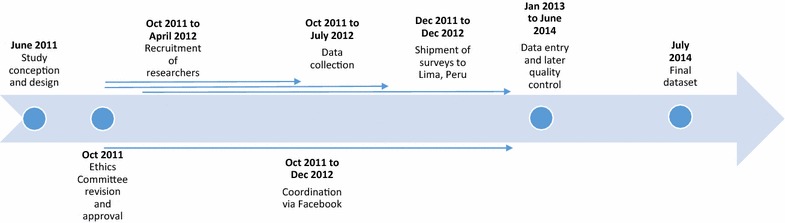
Timeline for the evaluation of the profile and professional expectations of medical students from 11 Latin American countries: the Red-LIRHUS project

We trained all researchers in data-gathering skills so they could effectively carry it out at their respective schools. Each researcher was requested to obtain the rosters and schedules of classes that brought together all or most of the students from the required years, to enable data collection in those settings. Rosters were not obtained in approximately half of the cases. From October 2011 to July 2012 (roughly two academic semesters as it does not precisely correspond with the academic year for most of the subject universities), and with the permission of the course coordinator and the responsible teacher of the class, the researchers explained the project and distributed the survey to the students who accepted. The investigators remained in the classrooms to resolve any emerging concerns of the study subjects. Missing students were identified so as to locate them in another class. A student was definitely considered “missing” after three unsuccessful attempts to locate him or her. In 2012, all completed surveys were packaged and shipped by courier to Lima, Peru, to be digitized. Figure 2 shows a detailed timeline of the processes.

### Study subjects

Using available information, we estimated a total of 16,014 students in the first and fifth year in the 63 medical schools. Of them, 279 refused to take part in the research, 52 returned surveys blank or nearly blank (unusable) and 4441 were missing. A total of 11,573 completed surveys were obtained (Fig. 1). We then excluded the invalid surveys, most of which were partially completed (lacking any of our main outcomes). The final sample size for later analysis was 11,072. Table 2 shows the main characteristics of these subjects according to their year of study.

**Table 2:** Profile of the first- and fifth-year medical students from 11 Latin American countries included in the Red-LIRHUS project

Variables	Total	First-year	Fifth-year	p^c^
	n (%)	n (%)	n (%)	
Sociodemographic				
Age^a^	20.4 (3.0)	19.0 (2.0)	23.2 (2.3)	<0.001^d^
Male gender	5224 (47.3)	3365 (46.6)	1859 (48.6)	0.040
Married/cohabiting	364 (3.3)	153 (2.1)	211 (5.5)	<0.001
Have children	505 (4.6)	232 (3.3)	273 (7.2)	<0.001
Born in a rural area	598 (5.7)	412 (6.1)	186 (5.1)	0.045
Religious	9746 (88.0)	6447 (89.1)	3299 (86.1)	<0.001
Migrated for medical school	3524 (32.8)	2317 (32.9)	1207 (32.5)	0.695
Intermediate/advanced english language proficiency	5275 (47.6)	3282 (45.3)	1993 (52.0)	<0.001
National language-speaker^b^	887 (8.0)	595 (8.2)	292 (7.6)	0.265
Relative of a physician	5489 (49.8)	3635 (50.4)	1854 (48.6)	0.073
Relationship with someone living abroad	9182 (84.9)	6051 (85.7)	3131 (83.5)	0.002
University				
Country				<0.001
Bolivia	1618 (14.6)	1219 (16.8)	399 (10.4)	
Chile	606 (5.5)	302 (4.2)	304 (7.9)	
Colombia	1423 (12.9)	874 (12.1)	549 (14.3)	
Costa Rica	148 (1.3)	137 (1.9)	11 (0.3)	
Ecuador	1174 (10.6)	866 (11.9)	308 (8.0)	
El Salvador	94 (0.9)	51 (0.7)	43 (1.1)	
Honduras	990 (8.9)	556 (7.7)	434 (11.3)	
Mexico	201 (1.8)	136 (1.9)	65 (1.7)	
Paraguay	156 (1.4)	100 (1.4)	56 (1.5)	
Peru	3768 (34.0)	2422 (33.5)	1346 (35.1)	
Venezuela	894 (8.1)	575 (7.9)	319 (8.3)	
Capital city location	3441 (31.1)	2150 (29.7)	1291 (33.7)	<0.001
Publicly funded	7204 (65.1)	4652 (64.3)	2552 (66.6)	0.016
Medical career				
Have failed a class	3334 (30.9)	1790 (25.4)	1544 (41.2)	<0.001
Satisfied with medical career	9705 (88.7)	6393 (89.3)	3312 (87.6)	0.005
Total	11,072 (100)	7238 (65.4)	3834 (34.6)	

^a^ Mean and standard deviation

^b^ Native national or regional languages other than Spanish

^c^ Chi square test

^d^ Student’s T test

### Survey and outcome measurements

The survey was an anonymous, self-administered questionnaire previously tested in a pilot in a sample of Latin American medical students [17]. It assessed general data of the students and four main topical sections: (1) motivation for choosing medicine, (2) academic profile and professional expectations, (3) migration, and (4) perceptions of primary care.

Sociodemographic data included sex, age, marital status (single or married/cohabiting), birth place (city; urban or rural), university funding (private or public), religion (Catholic, Jehovah’s Witness, atheist/agnostic or other). We also asked about their English language skills (none, basic, intermediate or advanced). In the same way, skills in a language that was native in their country or region (e.g., Quechua in Peru; Guaraní in Paraguay) were assessed. We also evaluated relationships with someone (family or close friends) living abroad (yes or no) and having physicians as relatives (yes or no).

The English translation of the questionnaire is included herein as Additional file 1.

### Motivations for studying medicine

Motivations for studying medicine were assessed primarily through the Motivaciones para Estudiar Medicina (MEM-12) scale, which was validated in a Latin American student sample. Global internal consistency was high (0.74). The scale comprises 12 items from two components: (1) altruism, social conscience (6 items, 5–30 points; α = 0.80) and (2) social and financial status/position (5 items, 5–30 points; α = 0.71) [25].

Other important variables were also measured: age of decision to study medicine, external influence on choosing the career (parent, relative, schoolteacher or none). We also asked about prior admission to a hospital as a patient (yes or no) and the experience of taking care of a sick family member (yes or no) and if they thought these events influenced their career choice (yes or no) [26].

### Academic profile and professional expectations

Identifying an academic profile encompassed variables regarding performance in languages other than Spanish (language: basic, intermediate or advanced), participation in students’ scientific meetings (yes or no), intention to complete a thesis to graduate (yes or no), publications in scientific journals (yes or no), career satisfaction (yes or no) and having failed courses (yes or no).

Expectations about professional future included plans 10 years after finishing medical school. We inquired about plans to perform a master’s, doctoral or residency program (yes, no or not yet decided) and the first intended program option, expected number of jobs and expected salary (both numerical open questions). The main intended workplace was also addressed (hospital, health center, university or research center, administrative and policy-related organizations or others).

### Migration

Migration was assessed as per two different time frames and with several definitions because of the lack of a standardized definition. The first time frame corresponded to the migration to the medical school’s location. To generate this new variable, the university location was matched with variables referring to origin: (1) birth place, (2) location of completed high school and (3) having lived less than 5 years where the university is located (excluding the undergraduate study period).

The second time frame regarded migration for work. It was measured with the question, “Where do you expect to be working 10 years after finishing medical school?” The students were asked to indicate to what countries they were willing to emigrate, or what city if they were staying in their country. In either case—abroad or their country—some characteristics of the target area were obtained (country capital or provinces; urban or rural). To create a definition of migrant, this was matched with: (1) birth place, (2) location of completed high school and (3) location of completed medical school. For those who planned to emigrate, we asked about their intent to eventually return to their countries. When asked about their migration intent, students were also able to respond that they had not yet decided.

Other important variables contributing to migration-related issues were measured: English proficiency certification (FCE, TOEFL or IETLS or none), documentation to emigrate (passport, American/European visa or none), relatives living abroad (yes or no) and intent to enter a postgraduate program in a foreign country (yes or no).

### Primary care labor perspectives

An 11 item-scale validated in a sample of Latin American medical students [17] through a five-point Likert-type scale was used to evaluate perceptions on primary care labor. A simple sum of the item scores generated totals ranging from 11 to 55, which expressed the strength of perceptions in a favorable (lower score) or unfavorable (higher score) way. A global adequate internal consistency was found (α = 0.78). The scale was subdivided into three domains, representing specific perceptions about: (1) primary care physician (5 items, 5–25 points; α = 0.73), (2) primary care work itself (4 items, 4–20 points; α = 0.65) and (3) financial consequences for an individual in working in primary care (2 items, 2–10 points; α = 0.60) [27].

### Statistical analysis

Data were tabulated in Microsoft Excel (Microsoft Corporation, Redmond, WA, USA) and then subjected to a cleaning process. About 25% of observations were double-entered and matched. A randomized 10% of the other surveys was reviewed in search of concordance, which ultimately was good. We additionally assessed the descriptive analysis of each variable. Aberrant values, when found, were verified with the physical survey. Analyses were performed using STATA 14.0 (StataCorp LLC, College Station, TX, USA).

In the present study, categorical variables were described using absolute and relative frequencies; numerical variables were expressed in mean and standard deviation after normality testing. We compared characteristics between the first- and fifth-year students. Bivariate analysis was performed with Pearson’s Chi squared test and Student’s t test for categorical and numerical variables, respectively.

### Ethical approval

The study protocol was approved in 2011 by the Ethics & Research Committee of Instituto Nacional de Salud del Perú (Peruvian National Institute of Health) (223-2011-CIEI/INS). Additionally, permission was obtained from every participant institution’s ethics committee or equivalent competent authority (e.g., dean or equivalent).

For students who voluntary agreed to participate, informed verbal consent was obtained. The questionnaire was self-administered and included no fields that would enable personal identification.

### Costs and funding

This project had no funding grants. Total expenditure was approximately US$20,000. This amount only includes the salary of the data entry clerk; no other financial remuneration was paid. The principal investigator paid for the transport of the surveys to Lima and the digitation process. Individual researchers at their local centers paid for reproduction of the survey. This lack of funding may explain the delays in the stages of the study and deferral of the results’ publication.

## Discussion

### Sample of Latin American medical students

This study sought to evaluate characteristics and expectations of the Latin American health workforce in training. The final number of enrolled medical students who completed surveys valid for analysis was 11,072. Most of the study subjects were first-year students because of the normally expectable attrition rates in medical schools [28, 29]. The mean age of first-year subjects did not correspond to the average age at which students begin university studies in Latin America (17–19 years old, varying by country). This may be because the admissions process for medical students typically takes longer than for other courses, and may require certain preparation in pre-university institutions [17, 30, 31]. The slightly higher proportion of women reflects the growing representation of women in medicine [32]. As in other reports throughout the world, a small fraction of the students were from rural areas [8, 16] or were native speakers of a regional language other than Spanish [9]. Additionally, one out of two students were relatives of a physician. This is considerably higher when compared with other studies [6, 16]. A previous study found a similar result in a sample of recently graduated Peruvian physicians [9].

### What worked and did not work?

#### What worked?

- Collaborating with medical students as researchers was a risky but successful strategy [33].
- Using an online social network (Facebook) to recruit researchers was also an efficient approach [24].

#### What did not work?

- As expected, working without proper external funding prolonged times of execution and data entry. Consequently, longer times to publication were required.
- Working without a defined sample frame considerably limits representativeness.
- Having a paper-based survey led to three medical schools abandoning the study (Fig. 1). Even though this format was the best option given the lack of funding, electronic surveys through mobile devices might be taken into consideration for future studies.

### Strengths and limitations

The strengths of this study are in the wide scope of evaluation and the novel recruitment methods using social networking. We evaluated 63 medical schools, covering a wide range of Spanish-speaking countries through Latin America. These nations were reached using Facebook; as mentioned above, this was an efficient tool for contacting and recruiting, and for coordinating all aspects of research execution [24].

The present study has some limitations. At the time of the study, there was only one medical school in Honduras, which was included. For the other countries, we were not capable to evaluate at least one medical school in the capital city and one outside it, or at least one private and one public school. Only subjects from Peru, Colombia, Venezuela and Bolivia met these criteria for diversity. Because of this, and also taking into account that Peru was the only subject country to successfully complete a national census, the proportions obtained must be interpreted with caution.

We cannot extrapolate the results to all participating countries because not all medical schools there were assessed, with the exception of Peru, in which all 31 schools existing by 2011 were included. There is a certain potential for our information to be biased because of the high proportion of Peruvian subjects included. Additionally, not all collected surveys were valid for analysis. Another source of possible sample skew could be the use of a social networking service to recruit researchers leading to some restrictions preventing inclusion for certain medical schools in connecting in this virtual environment (e.g., rural or resource-limited schools). However, this Internet-based strategy enables recruitment and connection of a large number of schools and study subjects. Finally, the cross-sectional nature of our evaluation does not allow us to draw causal relations, but rather only associations.

Despite the limitations, this is, to our knowledge, the widest-ranging evaluation of Latin American medical students. Moreover, we reached a considerable sample size, and this aspect will be useful toward finding associations.

### Final reflections

Our data can be applied to provide indications of future workforce trends. This is an indispensable benefit, because no health improvements can be realized without ensuring available, skilled and motivated personnel [19]. Universities serve as quarries of the new generation of doctors. In that sense, academia represents a major stakeholder in solving the crisis. Transformative education planning must be aligned with governmental and international needs to counteract the erosion of health systems’ manpower [34–36].

## Additional file

**Additional file 1.** Questionnaire of the Red-LIRHUS project.

## Abbreviations

Red-LIRHUS: Grupo Colaborativo Latinoamericano para la Investigación en Recursos Humanos en Salud
FELSOCEM: Latin American Federation of Medical Students’ Scientific Societies
MEM-12: motivation for choosing medicine scale
