# Supplementary material for: Profile and professional expectations of medical students from 11 Latin American countries: the Red-LIRHUS project
Source: BMC Res Notes. 2017 Apr 20;10:159. doi: 10.1186/s13104-017-2479-y (PMC5397751; doi:10.1186/s13104-017-2479-y)
Supplement: Supplementary file 1 — Additional file 1. Questionnaire of the Red-LIRHUS project. [file 13104_2017_2479_MOESM1_ESM.doc]

**PROFILE AND PROFESSIONAL EXPECTATIONS OF LATIN AMERICAN MEDICAL STUDENTS**

Dear medical student:

This questionnaire is part of a research project and has the approval of the Comité de Ética en Investigación del Instituto Nacional del Perú. Your participation in this study is voluntary and by filling this questionnaire you give your consent to participate in the study. All data supplied is entirely confidential and anonymous. The results of this research will be published in a medical journal.

# Thank you for your cooperation.

**SECTION 1: GENERAL DATA:**

1. Sex: M ( ) F ( )
2. Age: _______________
3. Marital status: Single ( ) Married ( ) Concubine ( ) Other ________________
4. Do you have children? No ( ) Yes ( ) If YES, How many? __________
5. Place where you were born (city, country): ___________________ Area: Urban ( ) Rural ( )
6. Location (city, country): _________________________ Area: Urban ( ) Rural ( )
7. Place where you finished your secondary schooling (city, country): ____________________________________

Area: Urban ( ) Rural ( )

Funding: Public ( ) Private ( )

1. University: ________________________________________________ Funding: Public ( ) Private ( )
2. How long (years) have you lived in your current city/town, excluding the years spent studying in the university? _______________________

| 1. Year of study? | | First ( ) | Fifth ( ) | |  | |  | | |
| --- | --- | --- | --- | --- | --- | --- | --- | --- | --- |
| 1. Religion: | None/Atheist/Agnostic ( ) | | | Catholic ( ) | | Jehovah’s witness ( ) | |  |  |

Other ( ), which one________________________________________

1. Do you have any relatives in the medical field:

No ( ) Yes ( ) Who? *Parents/brothers-sisters ( ) Grandparents/uncles-aunts/cousins ( )*

1. Do you have any relatives who are health professionals (non-medical):

No ( ) Yes ( ) Who? *Parents/brothers-sisters ( ) Grandparents/uncles-aunts/cousins ( )*

1. Place where your parents were born:

Father: Country__________________/ City_______________ Area: Urban ( ) Rural ( )

Mother: Country_________________/ City________________ Area: Urban ( ) Rural ( )

1. Indicate the maximum education level (instruction) reached or completed by your parents:

| **Father:** | No formal education | ( ) |  | **Mother:** | No formal education | ( ) |
| --- | --- | --- | --- | --- | --- | --- |
|  | Primary | ( ) |  |  | Primary | ( ) |
|  | Secondary | ( ) |  |  | Secondary | ( ) |
|  | Technician/Trade | ( ) |  |  | Technician/Trade | ( ) |
|  | University | ( ) |  |  | University | ( ) |
|  | Postgraduate | ( ) |  |  | Postgraduate | ( ) |

1. Home/First Language: ________________________________

Which language(s) do you know besides Spanish?

English: No ( ) Yes ( ) Level: Basic ( ) Intermediate ( ) Advanced ( )

Other(s): No ( ) Yes ( ) Level: Basic ( ) Intermediate ( ) Advanced ( )

Native: No ( ) Yes ( ) ____________________ Level: Basic ( ) Intermediate ( ) Advanced ( )

____________________ Level: Basic ( ) Intermediate ( ) Advanced ( )

1. Have you ever applied for any English certification examinations such FCE, TOEFL, IETLS, and/or Cambridge?

Yes ( ) No ( )

Have you attained any of them? Yes ( ) No ( )

1. Do you have a passport? Yes ( ) No ( )
2. Do you have an American or European VISA? Yes ( ) No ( )
3. Do you have family (grandparents, parents, siblings, uncles, cousins) living abroad? Yes ( ) No ( )
4. Do you have close friends living abroad? Yes ( ) No ( )
5. Who do you live with?

Alone ( ) Partner ( ) Parents/Brothers-Sisters ( ) Friends ( ) Other relatives ( )

1. Is currently someone financially depending on you? Yes ( ) No ( )
2. Currently, do you have a paid job? Yes ( ) No ( )
3. Have you ever done a hospital rotation abroad? Yes ( ) No ( )

**SECTION 2: MOTIVATIONAL AND INFLUENTIAL REASONS TO STUDY MEDICINE:**

1. Age you first thought to study medicine: _______ years
2. Age you decided to study medicine: _______ years
3. Age you finished high school: _______ years
4. Age you started medical school: _______ years
5. Who was/were the most influential in your decision to study medicine? (Choose only **ONE** answer)

Parents ( ) Relatives ( ) Teachers ( ) Nobody, I chose by myself ( ) Others ( ) ____________________

1. Before applying to medical school, you enrolled: In an academy ( ) In a pre-university program ( ) Did not attend to any program ( ) Other ( )_____________________
2. Have you studied a career before medical school: Yes ( ) No ( ) *go to question 34*

Finished the career: Yes ( ) No ( )

Institution: Technical ( ) University ( )

At the same university Yes ( ) No ( )

1. Rate how much you agree or disagree with the following statements: (Write an “X” in the correct box):

| **I decided to study medicine because…** | **Totally disagree** | **Disagree** | **Neutral** | **Agree** | **Totally agree** |
| --- | --- | --- | --- | --- | --- |
| … I want to contribute to a better society. |  |  |  |  |  |
| … I want to constantly progress |  |  |  |  |  |
| … I can work with people. |  |  |  |  |  |
| … I can accomplish my dreams. |  |  |  |  |  |
| … I can earn a lot of money. |  |  |  |  |  |
| … I can help others. |  |  |  |  |  |
| … I will be respected by others. |  |  |  |  |  |
| … I will have economic safety. |  |  |  |  |  |
| … I can express/show values that could be used as a model. |  |  |  |  |  |
| … I will have a stable job. |  |  |  |  |  |
| … I will acquire a high social status. |  |  |  |  |  |
| … It will give me fame and recognition. |  |  |  |  |  |

1. When you decided to study medicine, you thought… (Choose only **ONE** answer)
   - - - Medicine is the only career that will satisfy me. ( )
       - Medicine is one of many careers that will satisfy me. ( )
       - Medicine may not be a career that will satisfy me. ( )
2. Have you ever been admitted to the hospital before medical school? Yes ( ) No ( )

*Do you consider that admission to have influenced your decision to study medicine?* Yes ( ) No ( )

1. Before medical school, have you ever taken care of a very sick family member? Yes ( ) No ( )

*Do you consider that situation to have influenced your decision to study medicine?* Yes ( ) No ( )

**SECTION 3: CURRENT SITUATION:**

1. Have you ever attended to any student scientific meeting (national or international)? Yes ( ) No ( )

*Have you authored a research paper presented?* Yes ( ) No ( )

*Have you orally presented your work?*  Yes ( ) No ( )

1. Have you done extracurricular research? Yes ( ) No ( )
2. Do you have at least one publication in a scientific journal? Yes ( ) No ( )
3. Have you ever been a teaching assistant (at medical school)? Yes ( ) No ( )
4. Do you have to write a thesis to graduate? Yes ( ) No ( )
5. Have you failed any subjects during your medical studies? Yes ( ) No ( )
6. Currently, are you satisfied studying medicine?

| Very satisfied ( ) | Satisfied ( ) | Neutral ( ) | Unsatisfied ( ) | Very unsatisfied ( ) |
| --- | --- | --- | --- | --- |

1. Have you ever thought of changing your career? Yes ( ) No ( )
2. *You consider the salary of a doctor in your home country as:*

| Very good ( ) | Good ( ) | Enough ( ) | Limited ( ) | Insufficient ( ) |
| --- | --- | --- | --- | --- |

1. Do you know the average salary of a doctor in your home country? Yes ( ) No ( )

If YES, approximately how much? ______________ $(US Dollars)

1. Do you think that salary is adequate? Yes ( ) No ( )
2. Do you currently feel admiration for a doctor? Yes ( ) No ( ) go to the next question

Now, please answer regarding the doctor you admire the most:

This person is: Male ( ) Female ( )

Is he/she a university teacher? Yes ( ) No ( )

Is he/she is a researcher? Yes ( ) No ( ) I dont know ( )

Workplace: Hospital ( ) Health center ( ) Other ( ) _____________

Would you like to be like him (her)? Yes ( ) No ( )

**SECTION 4: FUTURE CAREER INTENTIONS:**

1. After finishing your medical education, are you PLANNING to enrol in a medical specialization (residency) program? Yes ( ) No ( ) Not yet decided ( )
2. If applicable, which specialization are you PLANNING to apply to? (Mention only **ONE**) ___________________________________
3. If applicable, where are you PLANNING to do your medical specialization (residency) program? :

Home country ( )

Abroad ( ) which country? *_____________________________________*

1. Ten years after finishing medical school, do you plan to have finished a Master’s program?

Yes ( ) Not yet decided ( ) No ( ) go to question 54

1. Where are you PLANNING to do this Master’s program?

In your country ( ) which city? ______________________________

Abroad ( ) which country? ______________________________

1. Do you plan to do a PhD program? Yes ( ) Not yet decided ( ) No ( ) go to question 56
2. Where are you PLANNING to do this PhD program?

In your country ( ) which city? ______________________________

Abroad ( ) which country? ______________________________

1. Ten years after finishing medical school, do you plan to have completed any training abroad (fellowship, rotation, specialty, master’s or doctoral programs*): Yes ( ) No ( )*
2. 10 years after completion of your medical education, are you PLANNING to practice medicine in: (Answer each one of the following options)

In another country Urban area ( ) Rural area ( ) Which cuntry? ________________________

Your country’s capital Urban area ( ) Rural area ( ) Which city? __________________________

Your country’s provinces Urban area ( ) Rural area ( ) Which city? __________________________

I have not decided yet ( )

1. At some point, are you PLANNING to return to your home country to practice medicine there?

Yes ( ) No ( ) Do not know ( )

1. How long after completion of your medical education, are you PLANNING to return to your country? _____ years
2. Ten years after finishing medical school, where do you plan to be working (as main workplace; check only one):

( ) Hospital, clinic, specialized institute

( ) Health center, health post (primary care facility)

( ) University, research center

( ) Non-welfare organizations linked to health (Ministry of Health, PAHO, NGOs, etc)*.*

( ) Other (specify*):__________________________________*

1. Ten years after finishing medical school, do you plan to be working at (how many jobs at once):

a) A single job b) Two jobs c) Three jobs d) More than three jobs

1. Ten years after finishing medical school, you plan to have a monthly income of approximately:

$ ____________ (US dollars)

1. 10 years after completion of your medical education, are you PLANNING to continue your professional development by: (*Put an X where it corresponds*):

|  | **Very likely** | **Likely** | **Unlikely** | **Nothing likely** |
| --- | --- | --- | --- | --- |
| a) Working in a hospital |  |  |  |  |
| b) Working in a primary care facility |  |  |  |  |
| c) Working in a research program |  |  |  |  |
| d) Working in public health & epidemiology |  |  |  |  |
| e) Working as a university lecturer |  |  |  |  |
| f) Working in healthcare management/administration |  |  |  |  |
| g) Working in private/own consultation |  |  |  |  |

1. 10 years after completion of your medical education, are you PLANNING on:
2. Being Married Yes ( ) No ( ) Not decided yet ( )
3. Having children Yes ( ) No ( ) Not decided yet ( )
4. Buying a house/apartment Yes ( ) No ( ) Not decided yet ( )
5. Buying a car Yes ( ) No ( ) Not decided yet ( )
6. Working in a government position Yes ( ) No ( ) Not decided yet ( )

**SECTION 5: PERCEPTIONS ABOUT PRIMARY CARE:**

*For this research, we define as primary care physicians all doctors who work in any primary care facility (health posts, health centers) either in rural or urban areas in your home country or abroad; regardless of any specialty/residence if trained****.***

|  | **Mark with an X, as appropriate** | **Strongly Disagree** | **Disagree** | **Indifferent** | **Agree** | **Strongly Agree** |
| --- | --- | --- | --- | --- | --- | --- |
| 66 | *Do you think a doctor working in primary care has less prestige in society than a doctor who works in a hospital?* |  |  |  |  |  |
| 67 | *Do you think that a doctor working in primary care did not have any other options?* |  |  |  |  |  |
| 68 | *Do you think that a doctor working in primary care is less academically prepared than a doctor who works in a hospital?* |  |  |  |  |  |
| 69 | *Do you think that a doctor working in primary care has a lower income than a doctor who works in a hospital?* |  |  |  |  |  |
| 70 | *Do you think that a doctor working in primary care has a lower status within the medical profession, compared to a doctor who works in a hospital?* |  |  |  |  |  |
| 71 | *Do you think that if you worked in primary care in your country, you would not be able to meet your economic needs?* |  |  |  |  |  |
| 72 | *Do you think that primary care labor is a transition period between finishing medical school and the specialty (residence)?* |  |  |  |  |  |
| 73 | *Do you think that in primary care, physicians see uninteresting cases compared to hospital activities?* |  |  |  |  |  |
| 74 | *Do you think that primary care labor is a routine compared to hospital activities?* |  |  |  |  |  |
| 75 | *Do you think that primary care labor is llimited compared to hospital activities?* |  |  |  |  |  |
| 76 | *Do you think that the academic training you received at medical school is oriented towards hospital-activities rather than primary care labor?* |  |  |  |  |  |

Thank you for your time and cooperation.
